# Cognitive screening assessment in Thai older adults: a prospective study of the reliability and validity of the Abbreviated Mental Test

**DOI:** 10.1108/jhr-02-2020-0049

**Published:** 2021-02-18

**Authors:** Kamonthip Tanglakmankhong, Benjamin M Hampstead, Robert J Ploutz-Snyder, Kathleen Potempa

**Affiliations:** Research, Boromarajonani College of Nursing Udonthani, Udonthani, Thailand; Psychiatry, University of Michigan, Ann Arbor, Michigan, USA; School of Nursing, University of Michigan, Ann Arbor, Michigan, USA; School of Nursing, University of Michigan, Ann Arbor, Michigan, USA

**Keywords:** Cognitive dysfunction, Screening tool, Elderly, Thailand

## Abstract

**Purpose –:**

The purpose of this paper is to examine the reliability and validity of the Abbreviated Mental Test (AMT) and the agreement with the Mini-Mental State Examination (MMSE).

**Design/methodology/approach –:**

This cross-sectional study included 446 older adults who were recruited by cluster sampling from 200,481 adults aged more than 60 years. For each participant, the AMT was administered by village health volunteers and, on a separate day, by a trained professional who also administered the MMSE. Descriptive statistics, Bland and Altman levels of agreement, and Receiver Operator Curves (ROCs) were used to analyze data.

**Findings –:**

Administration of the AMT by village health volunteers during the annual health screening found cognitive impairment in only 1.12% of the sample. When the AMT was given to these same individuals by trained professionals, the rate of cognitive impairment was almost 24 times greater. Two items in the Thai AMT may require modification due to markedly elevated failure rates. At the cut score of 8, the sensitivity and specificity of the AMT relative to the MMSE were moderate (78.83 and 66.67%, respectively). The degree of agreement between AMT and MMSE was 0.49 (*p* < 0.001) and the correlation between the difference scores and the mean is exceptionally low (0.048).

**Originality/value –:**

Reliable and valid cognitive screening assessment requires the administrator to be well trained and the tools to be appropriate for the population. Although AMT is short and easy for a nonprofessional to administer, some items were not suitable due to construct validity and contextual issues.

## Introduction

As with many other countries, the proportion of older adults is dramatically increasing in Thailand [1–3]. This rapid demographic transition is associated with a rise in the number of people living with dementia. The 5th Thai National Health Examinations Survey in 2014 found that the number of older adults with dementia was 8.1% (6.8% male and 9.2% female) [4]. Prasartkul *et al.* [3] reported the number of older adults with dementia in Thailand was 617,000 people in 2016 (206,000 male and 411,000 female). An Alzheimer’s Disease International report [5] predicts that the number of dementia cases in Thailand will reach more than 1.11 million by 2030 and 2.07 million people by 2050.

Cognitive decline is considered an early precursor of dementia. Cognitive impairment is one of the 10 crucial screening indicators for adults 60 years of age and older in Thailand and has been reported to the National Health Data Center since 2016. The national goal of screening 100% of older adults for cognitive impairment is rapidly being realized as the percentage of screened older adults increased from about 14.1% in 2016 to 72% in 2019. The value of these screenings is entirely dependent on the reliability and validity of the two screening measures approved by the Institute of Geriatric Medicine of the Thai Ministry of Public Health: The Abbreviated Mental Tests (AMT) and the Mini-Mental State Examination (MMSE) [6]. The standard cognitive screening process involves an initial assessment with the AMT that is conducted by lay village health volunteers who are community members and help professional health personnel in primary care units to implement health programs in the villages. An AMT score below 8 (maximum possible score of 10) is suggestive of cognitive impairment and triggers a secondary assessment using the MMSE administered by nurses or public health professionals in a primary care unit [6]. The MMSE Thai 2002 has shown adequate sensitivity and specificity for cognitive decline in Thai populations [7]. In addition to the MMSE [7], several cognitive screening tests have been evaluated in Thailand including the Montreal Cognitive Assessment-Basic (MoCA-B) [8], Thai Mental State Examination (TMSE) [7], the Thai version of Mini-Cog [9] and the Chula Mental Test [10].

We used data from the Thai national database and were the first, to our knowledge, to examine the performance of the AMT and the MMSE using a retrospective design with the AMT and MMSE taken at different times, by different people and under different conditions [11]. The results of that study revealed a poor agreement between the AMT and MMSE that likely emerged from two factors. First, multiple AMT items failed at an alarming rate (e.g. >50% of older adults), suggesting population-specific modifications are needed. Second, and the focus of the current manuscript, the AMT is administered by village health volunteers whereas the MMSE is administered by trained professional healthcare workers. Thus, the current prospective study had two primary objectives. First, we examined the effect of training by comparing AMT scores for the same individual when administered by village health volunteers vs trained professional healthcare workers. Second, we re-evaluated the relationship between AMT and MMSE scores when both are administered by trained professional healthcare workers.

## Methodology

### Sample and procedure

The routine national screening AMT results were acquired between October and December 2018 using village health volunteers as testers. These data were contrasted with the prospective re-assessment of AMT results in this same group from January to April 2019, when the test was administered by trained nurses and public health professionals in primary health care units. We did a cross-sectional study of a face-to-face screening survey database with 200,481 older adults from Udon Thani, Thailand, who were 60 years of age or more in 2018. Based on a priori power calculations, we determined that a sample size of 134–535 would provide 80–90% power with an alpha of <0.05. However, we followed Bujang and Adnan’s [12] conclusions that a minimum sample of 300 is often sufficient to evaluate sensitivity and specificity for most screening and diagnostic tests. A total sample of 446 older adults were recruited to allow for up to 30% attrition in order to meet our sample goals. The inclusion criterion was to be a Thai 60 years of age or more who were screened with the AMT between October and December of 2018 and agreed to be re-assessed from January to April 2019. Older adults exhibiting aphasia, deafness, unconsciousness, refusal to participate or severe illness were excluded from the study.

Using cluster sampling, 446 older adults were recruited from two primary health care units in one district in Udon Thani, Thailand. All data were anonymized prior to analysis.

Using a guide table to estimate the minimum sample size required for a screening test, and diagnostic studies of Bujang and Adnan in 2016 [12]), the table was derived from the formulation of sensitivity and specificity test using Power Analysis and Sample Size software based on the desired type I error, power and effect size. The prevalence of the mild cognitive impairment is estimated to be 20% [13], a minimum sample size of 535 subjects were required to achieve a minimum power of 80% (actual power = 81.9%) to detect a change in the percentage value of sensitivity from 0.80 to 0.90, based on a target significance level of 0.05 (actual *p* = 0.040). This minimum sample size was also sufficient to detect a change in the value of specificity from 80 to 90% which required a minimum sample of 134. Therefore, the sample size ranged from 134 to 535 subjects. Bujang and Adnan suggested that a sample of a minimum of 300 subjects is often sufficiently large to evaluate both the sensitivity and specificity of most screening or diagnostic tests [12].

Each of the professional healthcare workers was required to undergo training on the proper administration and scoring of the AMT and MMSE as well as in data entry procedures prior to the start of the re-assessments in January 2019. Our trained professionals collected informed consent from all-volunteer participants before data collection. The identity of each participant was kept confidential and the participant was informed that no personal identifiers would be recorded on the questionnaires. Once they had signed the consent form, the participants were screened using both the AMT and MMSE screening tests. The measures were coded, and the contact information was kept separately in a secure location to protect identities. Anonymous data were then entered into our research database for subsequent analysis.

### Measures

#### The Thai Abbreviated Mental Test (AMT)

The Thai Abbreviated Mental Test (AMT) has been translated by the Department of Medical Service, Ministry of Public Health, Thailand, against the original version of the AMT in English by Hodkinson [14]. It includes 10 questions that examine orientation in time, person and place, attention and recent memory, remote memory and general knowledge. The administration of the test takes between 3 and 5 min to complete [15]. Each question scores 1 point, and the total score ranges from 0 (no correct answer) to 10 (10 correct answers). The English version had been validated with 168 older adult patients in the Royal London Hospital Medical College. The best cut-off score was 8 with a sensitivity of 91% and a specificity of 75%. The reliability coefficient, Cronbach’s alpha, based on the internal consistency was 0.90 [16]. According to Thai national guidelines, the AMT scores less than 8 suggest abnormal cognitive function and triggered a secondary assessment using the MMSE administered by nurses or public health professionals in a primary care unit [6]. Once the case is identified, the case then should be referred or further assessed for getting the right diagnosis.

#### The Mini-Mental State Examination Thai version (MMSE-Thai 2002)

The Mini-Mental State Examination Thai version (MMSE-Thai 2002) was validated against the original version of the MMSE in English [17]. It is a 30-item questionnaire that is used extensively in clinical and research settings to measure and screen for cognitive impairment. Administration of the test takes between 5 and 10 min and examines functions including registration, attention and calculation, recall, language, ability to follow simple commands and orientation. The MMSE-Thai 2002 was translated and validated with 272 older adults in clinical and community settings in five regions all over the country using the criteria for the diagnosis of dementia as found in Diagnostic and Statistical Mental Disorders 4th edition (DSM-IV) [7]. The cut-off scores on the MMSE-Thai 2002 were tested and adjusted based on participants’ educational levels. The cut-off scores were 14 for older adults who are illiterate or have not completed elementary school, 17 for elementary education and 22 for higher than primary levels of education [6, 7].

### Data analysis

All statistical analyses were conducted using Stata (v.16) software [18]. Both AMT and MMSE-Thai 2002 data showed non-normal distributions, as indicated by significant Shapiro–Wilk (S–W) tests of normality. Descriptive statistics for these continuously scaled variables, therefore, include means, standard deviation (SD) and also medians, and interquartile range (IQR). We used Bland and Altman levels of agreement analytic methods and plots to characterize agreement between the AMT and MMSE scores after standardizing both scores to a percentage correct, and Lin’s concordance to characterize the association overall. Receiver operator curves (ROC) were created to evaluate how different cut-points on the AMT for a diagnosis of cognitive impairment performed relative to the gold standard diagnosis of the MMSE-Thai 2002. Sensitivity, specificity, positive and negative predictive values, and ROC curves are reported.

### Ethical issue

The study was approved by the Institutional Review Board at Udon Thani Provincial Public Health Office on January 4, 2019 (UDREC 0862).

## Results

### Screening administration

Despite being required to perform the cognitive screening tests, 84.2% of professionals in primary care centers did not have experience with cognitive screening. After training, professionals were able to administer the cognitive screening according to the guidelines for every step except having a table or private room available to do the test. Anecdotal information from statements made by participants suggests that most older adults felt more comfortable and confident having the screening test administered by trained professionals rather than village health volunteers who participants felt to be not as professional in giving this test.

Screening using the AMT by village health volunteers during the annual health screening for older adults in Thailand from October to December 2018 found a cognitive decline in only 1.12% of those tested. In contrast, screening using AMT by trained professionals among the same older adult group found a cognitive decline in 26.28% of those tested, almost 24 times greater. Thus, the findings for objective 1 highlight the need for training in the proper use of these measures. For further data analysis described below, we used the results of the AMT screening done by the trained professionals from January to April 2019.

### Sample characteristics

The characteristics of the sample are shown in Table 1. The average age was 69.02 years (SD = 6.33; median = 68, IQR = 64–73), and the majority (81.84%) had an elementary education. Cognitive impairment, screening using the AMT by professionals, was 26.68% in the entire sample. The average score of AMT was 8 (mean 8.08, SD 1.39; median 8.00, IQR 7.00–9.00). According to the MMSE-Thai 2002 screening test, 12.10% of the sample was cognitively impaired. Age-related cognitive decline was found in both AMT and MMSE. Males had higher rank scores of AMT and MMSE than females. There was a difference in AMT and MMSE scores between education levels. Higher AMT scores were associated with higher educated (*p* < 0.001), younger males (*p* < 0.05).

### Reliability and validity of the Abbreviated Mental Test (AMT)

For 446 adults tested, the median AMT was 8.00 (interquartile range 7.00–9.00). The average AMT score was 8.08 (±1.39). Only a small percentage of older adults in both groups, impaired and unimpaired, answered item number 8 on the AMT (“year of the great sorrow”) correctly, 3.36 and 19.27%, respectively. Another item that older adults had difficulty responding to was item 4, “the current year” (73.38 %). On the original English version of the AMT, the two-step that most older adults could not answer was the recall of address (28%) (Table 2).

The median MMSE-Thai score was 23 (interquartile range 22.00–26.00). The average MMSE-Thai score was 22.87 (±4.47). The sample population cognitive impairment, as defined by MMSE-Thai, was 12.10%. The items that most older adults could not answer correctly, and thus were nondiscriminatory regarding cognitive ability, were questions about attention, calculation and recall. Only 5.61% of the sample completed all serial subtractions accurately and only 31.84% could recall all three words correctly.

We evaluated item internal consistency on our Thai AMT data using Cronbach’s alpha and present our results (by item) alongside a prior Iranian study [19] (Table 3). Cronbach’s alpha for the 10 AMT items was 0.65. Our item-correlations with the overall factor score were clearly much lower than the 2017 Iran study [19]. This result suggested that more research is necessary in order to understand the potential reasons for the relatively poor fit of items in our data with older Thai adults.

### Agreement between AMT and MMSE scores

**Figure 1.**
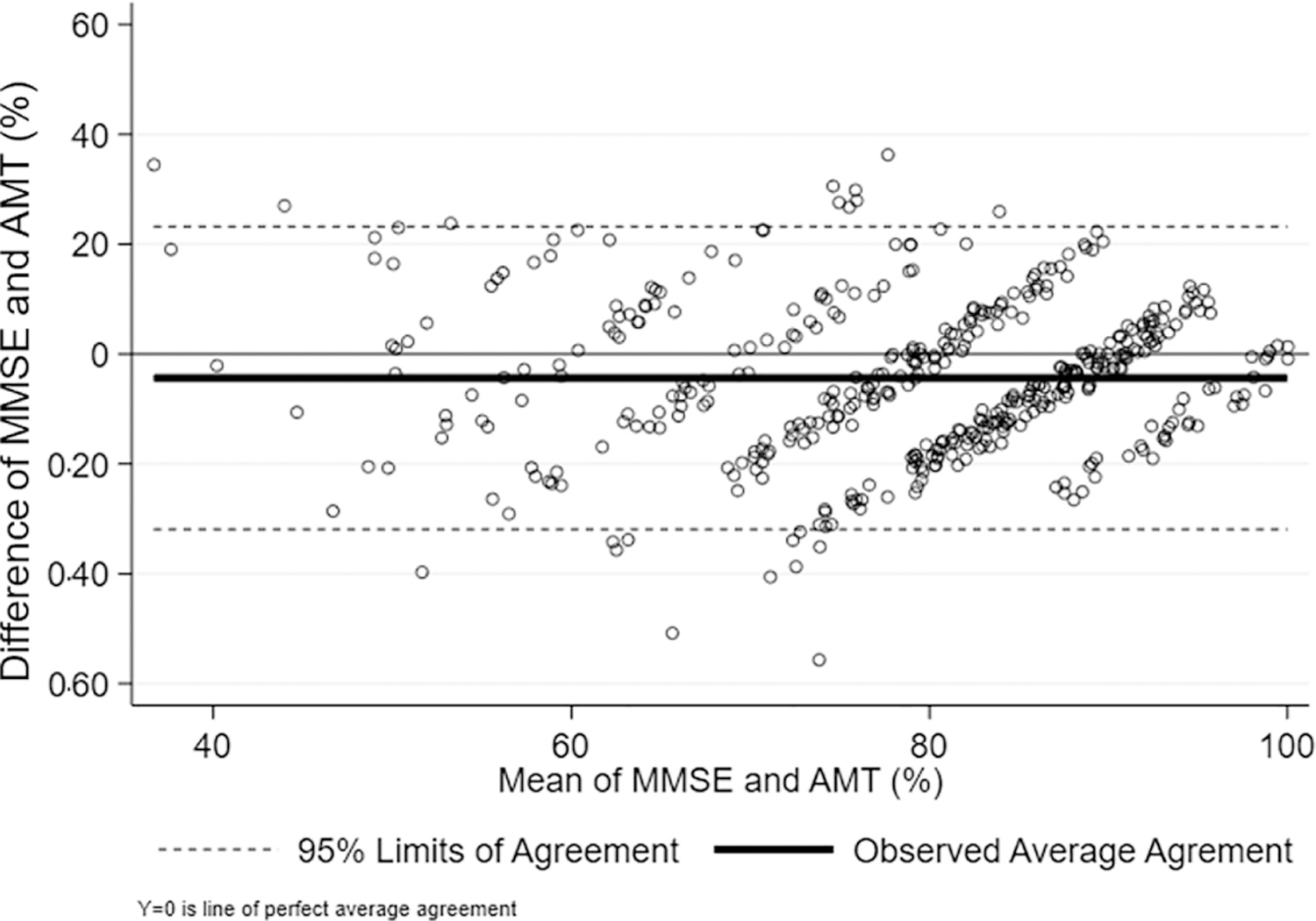
Bland–Altman plot between AMT and MMSE %

We converted AMT and MMSE raw scores to percent correct out of the total in order to evaluate the agreement between them. Bland and Altman levels of agreement are shown in Figure 1, with the mean (SD) difference score between the percent correct as indicated by the two measures of −4.35 (±14.06) and 95% levels of agreement of 31.92 and 23.21. The mean percent of AMT was higher than the mean percent of MMSE. Lin’s concordance correlation coefficient was rho = 0.49 (SE = 0.04; *p* < 0.001) indicating a nonchance association, though this may be artificially optimistic given the large sample size (*n* = 446). Still, the correlation between the difference scores and the mean is exceptionally low (0.048), suggesting that within the ranges observed here, there is no score-dependent bias in association [20]. We considered MMSE-Thai 2002 scores as the “ground truth” of cognitive impairment since, at this time, there are few specialty clinics (e.g. memory disorders clinics) and no biomarkers or consistently used criteria for dementia employed in the primary care setting in Thailand.

**Figure 2.**
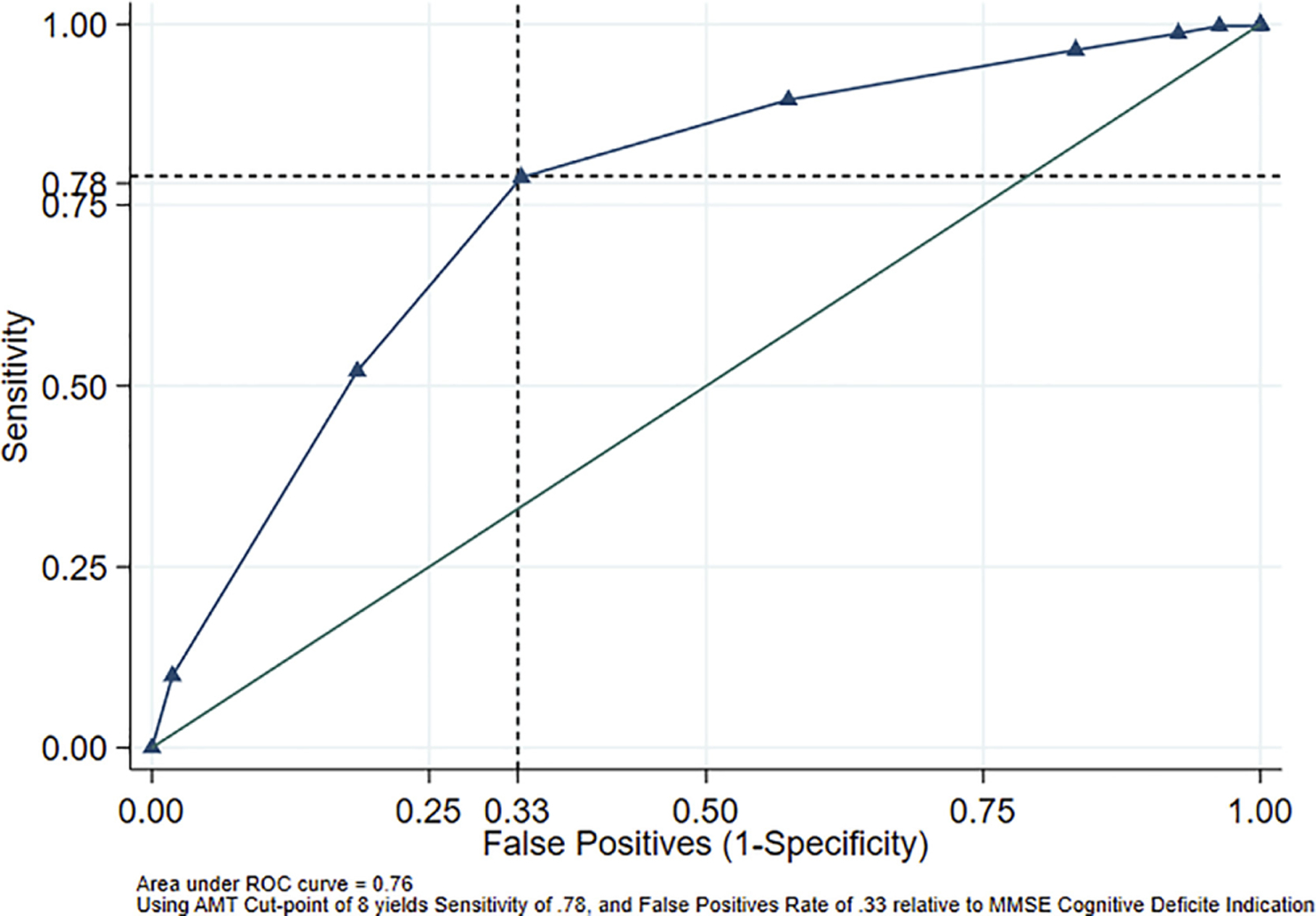
The receiver operating characteristic curves (ROC) of AMT

In practice, the AMT is currently used alone in Thailand to detect early cognitive impairment. As such, the sensitivity, specificity, positive and negative predictive values of AMT at different cut-off points for determining cognitive impairment are shown in Table 4. At the recommended AMT cut point of 8, the sensitivity was 78.83%, the specificity was 66.67% (Table 4). However, it is always desirable to have a test that is both high sensitivity and high specificity, our data suggest that the traditional cut-off of 8 in the AMT does not clearly classify a person as being at risk of cognitive decline. The larger values of area under the ROC curve (AUC) suggest the better overall performance of a diagnostic test [21], the values of AUC in Figure 2 is moderate (AUC = 0.76).

## Discussion

As shown in the cognitive screening results, the proportion of older adults identified as having cognitive impairment when the AMT was administered by village health volunteers (1.12%) was 24 times lower than when it was administered by trained professionals (26.68%), and 11 times lower than that revealed by MMSE-Thai screening (12.10%). These findings demonstrate the necessity of test administration being performed by well-trained personnel given both the local and national ramifications of such under-identification. The percentage of adults with cognitive impairment as indicated with the MMSE-Thai compares favorably with that seen in other countries. The range of cognitive impairment by MMSE in multiple counties was around 1.2–38% (15). Incorrect answers on the MMSE-Thai in our sample mostly related to attention/calculation and recall. These results are similar to findings in most other countries [22].

Although several studies showed that the AMT has high sensitivity and specificity in older adults in other countries and languages [16, 23, 24], our study found that the Thai AMT cut-off score of 8 detected 78.83% of older adults with cognitive impairment (true positive) but 21.17% with cognitive impairment go undetected (false negative). High sensitivity is clearly important where AMT is used to identify the risk of dementia among the older adult population. While the AMT, when properly administered, yielded a proportionately greater number of people with cognitive decline than the MMSE-Thai 2002, erring on the side of greater sensitivity is important so that early identification, validation and intervention can be employed for those at risk for further decline and progress to dementia.

In addition to the necessity of training, our data suggest that individual AMT questions contribute to its suboptimal psychometric properties. The most poorly answered item in the original English-based version of AMT asked individuals to recall their address (Item 3), thereby suggesting that older adults had difficulty with short-term memory [16]. In contrast, our data from the Thai AMT, this item was modified to ask only about the test taker’s current address, a question that might detect a problem with the orientation to place instead of with attention and short-term memory [22]. Recall memory is an important task that is always found to be problematic in the earliest stages of the cognitive dysfunction associated with Alzheimer’s disease [25]. This item made us wonder whether the Thai AMT measures a theorized psychological construct of cognitive impairment since item 3 was proved to be one of the best combination items for separating normal and cognitive decline [16, 25, 26].

Another item that most participants performed poorly on in AMT was item 8 that asks about “the year of great sorrow on October 14” which happened in 1973 between university students and the military dictatorship in Bangkok, Thailand [27]. The very high percentage of incorrect responses for this item (around 85%) suggests that it is not suitable for the entire Thai population, especially those who live in rural areas and/or those with lower education levels. Our findings are not unique in this regard since a majority of participants were unable to answer a similar question in a Persian version [28], i.e. the year the Iran–Iraq war started; or another study that inquired about the year Second World War ended [16]. Adjustments to this item may be necessary based on the older adults’ experiences related to an important national, or even local, occurrence or situation. In the original version of AMT in the UK, item 8 asked about the years of First World War or II, and in the US, the item asked what year the 9/11 attack on the twin towers took place [29]. We recommend that a different commonly understood potentially local, regional or national event in Thailand should be evaluated for validity for item 8.

Because of these issues with two of the ten items on the AMT, we conclude that the overall test validity comes into question. Also, in our sample, the values of the area under the ROC curve, an indication of test accuracy, is 0.75, considerably less than the optimum value. In general, higher AUC values indicate better test performance. The possible values of AUC range from 0.5 (no diagnostic ability) to 1.0 (perfect diagnostic ability) [30].

## Conclusions

Our findings suggest that (1) the evaluation of cognitive impairment should involve trained professionals given the significant societal ramifications associated with a missed diagnosis, (2) the Thai AMT should be, at a minimum, modified and re-evaluated to produce a scale with better psychometric properties or reconsidered as a screening tool entirely and (3) the trained professionals should provide thorough training to village volunteers. Future studies should compare the results from such brief measures (e.g. AMT, MMSE) with a gold standard reference for the clinical diagnosis of dementia in Thai older adults, especially comprehensive neuropsychological testing.

## Figures and Tables

**Table 1.** Characteristic of the sample

Characteristics	*N* = 446	AMT mean (SD)	MMSE mean (SD)
Age: Mean (SD)	69.02 (±6.33)		
Minimum–maximum	60–87		
(1) 60–69	262 (58.74%)	8.30 (±1.24)	23.80 (±3.95)
(2) 70–79	152 (34.08%)	7.77 (±1.47	21.78 (±4.55)
(3) 80–89	32 (7.18%)	7.69 (±1.73)	21.15 (±4.67)
*Gender*			
(1) Female	320 (71.81%)	7.97 (±1.47)	22.79 (±4.34)
(2) Male	126 (28.19%)	8.34 (±1.09)	23.26 (±4.32)
*Education*			
(1) Not completed elementary school	21 (4.71%)	6.71 (±2.00)	19.00 (±4.47)
(2) Elementary school	365 (81.84 %)	8.02 (±1.32)	22.63 (±4.25)
(3) Higher than elementary education	60 (13.45 %)	8.90 (±1.00)	26.08 (±2.76)
*Cognitive impairment screening result*			
(1) Total		8.08 (±1.39)	22.87 (±4.47)
(2) Normal *N* (%)		327 (73.32%)	392 (87.90%)
(3) Abnormal *N* (%)		119 (26.68%)	54 (12.10%)

**Table 2.** Percent correctly answered, by item, on AMT among impaired (AMT <8) and nonimpaired group (AMT 8–10)

AMT	AMT <8 (*N* = 119) 26.62% Correct (%)	AMT 8–10 (*N* = 327) 73.38% Correct (%)	Total (*N* = 446) 100% Correct (%)	Total AMT from the original English version in 1974 Correct (%)
1 Age	85.71	96.94	93.95	60
2 Current time	79.83	98.78	93.72	72
*3 Address*	*94.96*	*99.08*	*97.98*	*28 (recall address)*
4 Current year	38.66	85.63	73.09	41
5 Current location	95.80	98.47	97.76	60
6 Recognition 2 persons	87.39	98.78	95.74	76
7 Date of birth	41.18	91.13	77.80	80
*8 Year of great sorrow on October 14*	*3.36*	*19.27*	*15.02*	*63 (Year of First World War)*
9. Name of King Rama IX	71.43	93.58	76.23	64 (Name of present monarch)
10. Count backwards from 20–1	60.50	96.02	86.55	62 (Count backwards from 20–1)

**Table 3.** The corrected item item-total correlations of Cronbach’s alpha values

AMT questions	Cronbach’s alpha		Remark
	Thai 2019 (*n* = 446)	Iran 2017 (*n* = 101)	
1 Age in years	0.66	0.88	
2 Current time	0.63	0.89	
3 Address	0.67	0.89	
4 Current year	0.63	0.91	
5 Current location	0.68	0.90	
6 Recognition 2 persons	0.65	0.89	
7 Date of birth	0.62	0.89	
8 Year of great sorrow on October 14	0.66	0.90	Year the Iran–Iraq war started
9 Name of King Rama IX	0.60	0.89	Name of current leader
10 Count backward from 20–1	0.62	0.88	
*Total*	*0.65*	*0.90*	

**Table 4.** Sensitivity, specificity, PPV and NPV of the AMT at different cut-off points

AMT cut-off point	Sensitivity (%)	Specificity (%)	PPV (%)	NPV (%)
2	100.00	0.00	0	0
3	99.74	0.00	87.9	0
4	99.74	3.70	88.3	66.7
5	98.72	7.41	88.6	44.4
6	96.43	16.67	89.4	39.1
7	89.54	42.59	91.9	35.9
8	78.83	66.67	94.5	30.3
9	52.04	81.48	95.3	19.0
10	9.95	98.15	97.5	13.1
